# Analysis of the Correlation Between Circulating Cell-Free DNA (CfDNA) Levels and Serious Abnormal Sperm Parameters

**DOI:** 10.3390/cimb48090911

**Published:** 2026-09-05

**Authors:** Oumar Konate, Modou Mamoune Mbaye, Bouchra El Khalfi, Bouchra Ghazi, Fatiha Elmellouli, Hasnae Zekhnini, Noureddine Louanjli, Abdelaziz Soukri

**Affiliations:** 1 Laboratory of Health, Environment and Biotechnology, Pathophysiology, Molecular Genetics and Biotechnology Team at the Ain Chock Faculty of Sciences, Hassan II University of Casablanca, Casablanca 20360, Morocco; oumar.k2017@gmail.com (O.K.); bouchraelkhalfi@gmail.com (B.E.K.); 2 Medical Analysis Laboratory, Reproductive Biology, Labomac, Casablanca 20100, Morocco; mbayeass87@gmail.com (M.M.M.); n.louanjli@gmail.com (N.L.); 3 Immunopathology-Immunotherapy-Immunomonitoring Laboratory, Faculty of Medicine, Mohammed VI University of Health and Sciences, Casablanca 82403, Morocco; bghazi@um6ss.ma; 4 Mohamed VI Center for Research & Innovation (CM6RI), Rabat 10100, Morocco; 5 Regional Laboratory for Analysis and Research Casablanca, National Office for Food Safety “ONSSA”, Rabat 10050, Morocco; fatihaelmellouli@gmail.com (F.E.); ha.zekhnini@gmail.com (H.Z.)

**Keywords:** cell-free seminal DNA, qPCR, male infertility, sperm characteristics

## Abstract

Cell-free DNA (cfDNA) fragments represent emerging biomarkers of major interest in the assessment of male fertility and the management of couples undergoing assisted reproductive technology (ART). Easy to quantify and present in most biological fluids, seminal cfDNA could reflect apoptosis and necrosis occurring within the male reproductive system. In this study, we compared cfDNA concentrations, measured by means of qPCR targeting the RNase P gene, in the seminal plasma of men with severe sperm quality abnormalities, including very severe asthenozoospermia (*n* = 25), total azoospermia (*n* = 25), very severe oligozoospermia (*n* = 25), and very severe teratozoospermia (*n* = 25), with those of normozoospermic controls (*n* = 25). Mean cfDNA concentrations were particularly elevated in men with azoospermia (5.47 ± 1.17 µg/mL) and very severe teratozoospermia (3.25 ± 1.21 µg/mL), compared with controls (1.96 ± 0.27 µg/mL). After adjustment for multiple testing, only azoospermia and very severe teratozoospermia remained significantly associated with increased cfDNA levels (Holm–Bonferroni adjusted *p* < 0.003), whereas the other phenotypes did not maintain statistical significance. These results suggest that seminal cfDNA is markedly increased in the most severe forms of spermatogenic impairment, potentially reflecting enhanced germ cell apoptosis or testicular cell involvement. Despite some limitations, including the modest sample size and single-center design, our observations support the value of cfDNA as a promising non-invasive biomarker of sperm quality. Larger multicenter and longitudinal studies are needed to validate its clinical relevance and determine standardized reference thresholds for clinical application.

## 1. Introduction

Nucleic acids have become essential biomarkers in the management of male infertility. Although testicular biopsy remains the gold standard for characterizing spermatogenesis in azoospermia, it is invasive, costly, and not suitable for routine use [1,2]. To overcome these limitations, liquid biopsy approaches have emerged as promising non-invasive strategies based on circulating nucleic acids such as cell-free DNA (cfDNA) and microRNAs [3,4,5,6]. cfDNA fragments are released during apoptosis or necrosis within the male reproductive system [7] and can be detected in several biological fluids, including blood, semen, and prostatic secretions, offering new diagnostic and prognostic perspectives [8,9].

Recent work has strengthened the role of seminal cfDNA as a biomarker of male reproductive dysfunction. Elevated seminal cfDNA concentrations have been associated with defective spermatogenesis, oxidative stress, increased sperm DNA fragmentation, and chromatin instability [10,11,12,13]. Moreover, seminal cfDNA quantification has recently been proposed as a potential predictor of sperm retrieval success in men with non-obstructive azoospermia (NOA) [3,14], suggesting clinical applicability in precision andrology. Beyond male fertility, cfDNA analysis has already demonstrated clinical utility in oncology, transplantation medicine, and infectious diseases as a powerful tool for non-invasive diagnosis and monitoring [8,9].

Despite increasing interest, available studies are still fragmented and often limited to a single phenotype or insufficiently stratified according to severity. To date, no study has simultaneously evaluated seminal cfDNA variations across the four most severe forms of spermatogenic impairment defined by the WHO 2021 criteria: total azoospermia, very severe oligozoospermia, very severe asthenozoospermia, and very severe teratozoospermia [15]. In addition, data from North African populations remain scarce despite the high burden of male infertility in this region.

Our study addresses a critical knowledge gap by analyzing the first Moroccan cohort using a standardized RNase P-based qPCR protocol. By revealing markedly elevated seminal cfDNA concentrations, particularly in azoospermia, this work highlights the potential of cfDNA as a non-invasive discriminant indicator between obstructive and non-obstructive azoospermia (NOA) [2,14,16], helping to avoid unnecessary testicular biopsies and improve personalized management strategies.

We therefore hypothesized that seminal cfDNA concentration reflects the severity of spermatogenic impairment and could discriminate the most severe phenotypes from normozoospermic controls.

The aim of this study was to evaluate the association between seminal cfDNA levels and severe sperm quality alterations to determine its potential clinical utility as a complementary biomarker in male infertility.

## 2. Materials and Methods

### 2.1. Study Population

This study was conducted at the Laboratory of Health, Environment and Biotechnology, Pathophysiology, Molecular Genetics and Biotechnology Team at the Ain Chock Faculty of Sciences, Hassan II University of Casablanca, and included a total of 100 semen samples collected as part of a male fertility assessment. Participants were divided into two groups: (i) a control group consisting of men with normal sperm parameters according to the 2021 WHO standards, and (ii) a pathological group comprising patients with at least one very severe impairment in a characteristic parameter of sperm quality.

All participants provided written informed consent before the use of their samples.

### 2.2. Inclusion Criteria

This study included men undergoing fertility assessment and was divided into two groups based on the parameters defined by the WHO 2021 criteria. The control group consisted of normozoospermic volunteers with a sperm count ≥39 × 10^6^/ejaculate, total motility ≥42%, vitality ≥54%, and normal morphology ≥4%. The pathological group included patients with severe alteration of at least one of the sperm parameters, including azoospermia (absence of spermatozoa after centrifugation), severe oligozoospermia (<5 × 10^6^/ejaculate), very severe asthenozoospermia (total motility between 0 and 10% one hour after ejaculation), or very severe teratozoospermia (normal morphology < 1%).

### 2.3. Exclusion Criteria

This study excluded participants with a clinically diagnosed varicocele, active urogenital infection, or recent fever (<3 months), as well as those with chronic diseases such as diabetes, obesity, cancer, or any other inflammatory condition. Men with hormonal abnormalities, excessive tobacco or alcohol consumption, or those who had received treatment with antioxidants or antibiotics within three months prior to sampling were also excluded.

### 2.4. Collection of the Sperm Sample

Semen samples were collected at the Medical Analysis Laboratory, Reproductive Biology, Labomac, Casablanca, Morocco, by masturbation after 3 to 5 days of sexual abstinence, in a sterile wide-mouth container, according to standard laboratory procedures. After ejaculation, the samples were kept at 37 °C to ensure complete liquefaction. This was checked regularly every 15 min, for a maximum period of 60 min, before any analysis or manipulation.

### 2.5. Sample Preparation

Once collected and completely liquefied, semen samples were analyzed according to standard laboratory protocol and World Health Organization standards [9]. They were then centrifuged at 500× *g* for 10 min to avoid cell lysis, followed by centrifugation at 10,000× *g* for 5 min to remove sperm and retain only seminal fluid containing free nucleic acid fragments. The samples were then stored at −80 °C until use. For this study, each sample was analyzed in duplicate.

### 2.6. Free DNA Recovery

For nucleic acid purification, we used the MACHEREY-NAGEL NucleoSpin^®^ kit. This is a highly efficient and rapid DNA purification kit for isolating DNA from biological fluids. The principle is very simple: the use of silica columns purifies the DNA while removing contaminants.

For each sample, we used a volume of 200 µL and performed three successive washes, with a final elution volume of 50 µL per sample. All of these operations were performed under sterile conditions to avoid any contamination of our samples.

### 2.7. Quantification of Cell-Free DNA

To quantify cell-free DNA, we performed real-time PCR on the Applied Biosystems 7500 Fast system, using SYBR GREEN fluorescence detection to detect amplified products. We chose to use primers targeting RNase P (primers F 5′ AGATTTGGACCTGCGAGCG 3′ and R 5′ GAAGCCGGGGCAACTCAC 3′), a highly conserved endoribonuclease present in all living cells. The amplification process followed a four-step temperature profile: initial denaturation at 95 °C for 10 min, followed by 35 cycles including denaturation at 95 °C for 25 s, annealing at 60 °C for 30 s, extension at 72 °C for 30 s, and fluorescence measurement at 84 °C for 8 s.

### 2.8. Standard Range and Internal Controls

We had to establish a standard range. To do this, we used human DNA at a standardized concentration, established by quantitative fluorescence measurement on the Thermo Fisher Scientific^®^ Invitrogen QubitTM analyzer. We then performed half-fold dilutions in cascade to obtain four concentration points: 0.5 µg/mL; 1 µg/mL; 2 µg/mL; and 4 µg/mL. From this range, we established a calibration line showing the maximum and minimum concentrations framing our RNaseP samples.

We had to work in duplicate for each well to avoid adding a positive control to each PCR plate. Thus, each well serves as a control for its duplicate. Furthermore, we combined the two values to obtain the final value by simple average.

Finally, for each plate, we chose to reserve one well as a negative control. Master Mix and pure water were added to these wells.

### 2.9. Sample Size Calculation

The sample size was determined a priori to compare, separately, each of the four “case” groups (azoospermia, very severe asthenozoospermia, severe teratozoospermia, very severe oligozoospermia) with the control group using a two-sided Mann–Whitney test (asymptotically approximated by the *t* test). Setting α = 0.05 and power = 80%, and based on pilot data indicating a minimum expected difference of 1 µg/mL of cfDNA between groups (i.e., a standardized effect size of approximately 0.8), the required sample size was *n* = 25 per group. This size was retained for each of the four case–control comparisons (5 groups in total). To account for the multiplicity of comparisons, the main analyses were prespecified by phenotype, and sensitivity analyses with Bonferroni adjustment (corrected α = 0.0125) were planned; in this setting, the power remains adequate to detect important effects.

### 2.10. Statistical Analysis

To control for the inflation of the first-order risk associated with multiple comparisons between the four phenotypes studied (azoospermia, very severe oligozoospermia, very severe asthenozoospermia, and very severe teratozoospermia) and the control group, we applied a multiplicity correction. The *p*-values from the Mann–Whitney tests were adjusted using the Bonferroni (m = 4) and Holm–Bonferroni (stepwise method maintaining control of the familial risk α) procedures.

Statistical significance was primarily interpreted using the Holm–Bonferroni method, which is more powerful while controlling the overall error rate, while Bonferroni *p*-values are reported for methodological transparency. Significance was set at α = 0.05. For correlations, we report Spearman coefficients (ρ) and their 95% CIs, with similar adjustments where necessary. Graphical representations display only adjusted significances.

All analyses were performed using GraphPad Prism version 10 software.

### 2.11. Ethical Aspects

The study protocol was approved by the UM6SS Biomedical Research Ethics Committee (reference: CE/UM6SS/09/23) on 25 July 2023. Written informed consent was obtained from all participants prior to enrolment. All data were anonymised and analysed in accordance with the principles of the Declaration of Helsinki (2013 revision).

## 3. Results

### 3.1. Seminal Characteristics of the Study Population

The sperm characteristics of the participants are presented in Table 1. The mean age did not differ significantly between the groups: 32.93 ± 1.55 years for normozoospermic controls, 35.15 ± 1.01 years for azoospermic patients, 33.83 ± 1.55 years for men with severe asthenozoospermia, 31.61 ± 1.45 years for oligozoospermics and 37.14 ± 0.55 years for teratozoospermics (*p* > 0.05).

Compared with controls (3.41 ± 1.25 mL), seminal volume was significantly decreased in the azoospermic (1.90 ± 0.54 mL) and oligozoospermic (1.54 ± 0.46 mL) groups (*p* < 0.05), while the asthenozoospermic (2.84 ± 0.96 mL) and teratozoospermic (2.08 ± 0.79 mL) groups did not show a significant difference.

Sperm concentration was 48.56 ± 2.34 × 10^6^/mL in controls. It was significantly reduced in azoospermic patients (0.08 ± 0.19 × 10^6^/mL), severely impaired in oligozoospermia (2.26 ± 0.31 × 10^6^/mL) and decreased in asthenozoospermic patients (32.32 ± 2.59 × 10^6^/mL) (*p* < 0.05), while teratozoospermic patients had a concentration of 41.64 ± 1.80 × 10^6^/mL.

Total motility was 48.07 ± 1.02% in controls. It was significantly impaired in the asthenozoospermic group (14.43 ± 9.35%) (*p* < 0.05), while it remained comparable to controls in the oligozoospermic (46.94 ± 1.26%) and teratozoospermic (43.80 ± 1.03%) groups. Azoospermic patients had zero motility.

Vitality was 61.02 ± 2.70% in controls, compared to 57.07 ± 0.87% in cases of severe asthenozoospermia and 55.39 ± 0.03% in oligozoospermic patients (*p* < 0.05). It was 55.85 ± 0.91% in cases of severe teratozoospermia and zero in azoospermic patients.

Finally, normal morphology was 5.30 ± 1.30% in controls and significantly reduced in cases of azoospermia (0%), and in teratozoospermic patients (2.07 ± 1.33%) (*p* < 0.05), while it remained similar to controls in the asthenozoospermic (5.30 ± 1.30%) and oligozoospermic (5.07 ± 0.92%) groups.

### 3.2. cfDNA Concentrations According to Groups

The cfDNA concentrations in seminal plasma are presented in Table 2. The normozoospermic control group had a mean value of 1.96 ± 0.27 µg/mL. In the azoospermic group, cfDNA concentrations reached 5.47 ± 1.17 µg/mL, with a significant difference compared to the controls (*p* < 0.001; after Bonferroni correction: significant). Patients with severe teratozoospermia also had significantly higher values (3.25 ± 1.21 µg/mL; *p* < 0.001; significant after Bonferroni). In contrast, the variations observed in subjects with severe asthenozoospermia (2.04 ± 0.21 µg/mL; *p* = 0.339) and severe oligozoospermia (2.11 ± 0.21 µg/mL; *p* = 0.046) did not reach the threshold of statistical significance after Bonferroni correction (corrected α = 0.0125).

After correction for multiple comparisons, only azoospermia (p_Holm < 0.003) and very severe teratozoospermia (p_Holm < 0.003) retained a statistically significant difference with controls. On the other hand, the differences observed in cases of very severe oligozoospermia (p_Holm = 0.092) and very severe asthenozoospermia (p_Holm = 0.339) no longer exceeded the significance threshold Table 3.

### 3.3. Correlations Between cfDNA and Parameters

**Figure 1:**
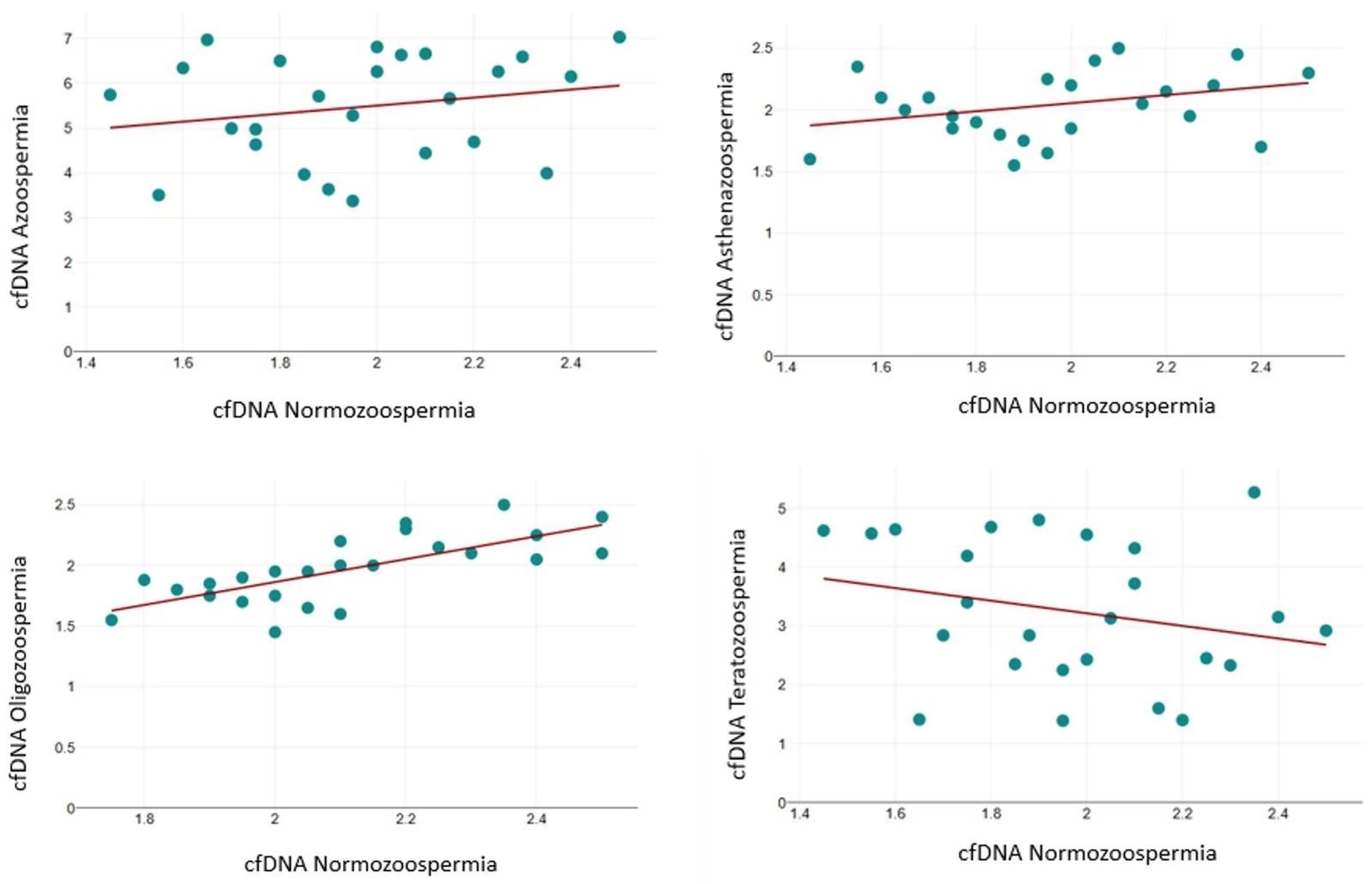
Correlations between samples of each pathology (**A**) Absence of spermatozoa in the ejaculate after centrifugation (azoospermia) (*n* = 25) (ρ = 0.21, *p* = 0.311); (**B**) low motility 1 h after ejaculation (between 0 10% a + b + C; asthenozoospermia) (*n* = 25) (ρ = 0.32, *p* = 0.119); (**C**) low sperm count (<10 million/mL; oligozoospermia) (*n* = 25) (ρ = 0.72, *p* = 0.001); and (**D**) high proportion of morphological anomalies (< 3%, > 1%; teratozoospermia) (*n* = 25) (ρ = −0.24, *p* = 0.241) and that of normozoospermic samples) (*n* = 25). Correlation coefficients (ρ).

Correlation coefficients (ρ) between cfDNA levels and sperm parameters are presented with their 95% confidence intervals. A significant positive correlation was observed in the very severe oligozoospermia group (ρ = 0.72; 95% CI: 0.50–0.85; *p* = 0.001) Figure 1C, suggesting that the increase in cfDNA may reflect the intensity of testicular apoptosis resulting in a decrease in sperm concentration.

In contrast, the correlations observed in the azoospermia (ρ = 0.21; 95% CI: −0.12–0.48; *p* = 0.311) Figure 1A, very severe asthenozoospermia (ρ = 0.32; 95% CI: −0.07–0.60; *p* = 0.119) Figure 1B and very severe teratozoospermia (ρ = −0.24; 95% CI: −0.55–0.13; *p* = 0.241) Figure 1D groups did not reach the significance threshold.

These results suggest that cfDNA release into seminal plasma does not solely reflect altered sperm concentration, but rather a set of pathophysiological processes affecting spermatogenesis, including increased germline apoptosis, chromatin condensation defects, and/or oxidative stress, particularly during the most severe forms of sperm dysfunction.

## 4. Discussion

This study provides novel insight into the clinical relevance of seminal cell-free DNA (cfDNA) as a biomarker of male infertility by simultaneously assessing the four most severe sperm abnormalities defined by WHO 2021 criteria (azoospermia, very severe teratozoospermia, very severe asthenozoospermia, and very severe oligozoospermia). cfDNA originates from cell apoptosis and necrosis within the male genital tract and represents an emerging molecular indicator of reproductive dysfunction [7,8]. Our findings show that azoospermic and very severe teratozoospermic men exhibited significantly elevated cfDNA concentrations, suggesting a strong association between severe spermatogenic failure and cfDNA release into seminal plasma. These results corroborate recent studies reporting that cfDNA reflects the degree of germ cell damage and defective spermatogenesis [3,4,17].

### 4.1. CfDNA and Pathophysiology of Severe Spermatogenic Impairment

The sharp increase in cfDNA in azoospermia supports the notion that, in cases of profound testicular dysfunction, massive apoptosis of spermatogonia and primary spermatocytes leads to extensive chromatin breakdown, contributing to increased nucleic acid shedding [2,16,18]. Importantly, this molecular signature persists even in the complete absence of spermatozoa, indicating that cfDNA could act as a non-invasive indicator of early maturation arrest. In addition, elevated cfDNA has been proposed as a potential discriminator between obstructive and non-obstructive azoospermia (NOA) by identifying molecular disruption upstream of sperm release [1,19,20,21].

Similarly, severe teratozoospermia was associated with high cfDNA levels, which aligns with evidence demonstrating that abnormal chromatin remodeling, protamine deficiency, and oxidative stress lead to decondensation and fragmentation of sperm nuclear DNA [11,13]. Therefore, cfDNA may reflect not only the quantity of gametes produced but also the quality of nuclear packaging and structural stability.

### 4.2. A Multifactorial Relationship Beyond Classical Semen Parameters

The absence of significant cfDNA elevation in very severe asthenozoospermia and oligozoospermia after corrected analyses suggests that motility decline alone does not imply major apoptosis-driven DNA release. The only significant correlation was found in very severe oligozoospermia, indicating that below a critical threshold of spermatogenic output, apoptosis becomes the dominant biological mechanism responsible for cfDNA release.

Taken together, these data indicate that cfDNA constitutes a global molecular indicator of spermatogenic integrity, beyond the scope of individual semen parameters such as motility or concentration.

### 4.3. Translational Implications: Toward Precision Andrology

From a clinical perspective, seminal cfDNA is a promising complementary tool to standard semen analysis for diagnostic and prognostic evaluation in reproductive medicine. The integration of cfDNA measurement into infertility work-ups could: (i) improve discrimination between NOA and post-testicular obstruction, potentially reducing unnecessary testicular biopsies [2,3,20], (ii) support decision-making regarding micro-TESE planning and ART strategies [1,4], (iii) help identify patients with high oxidative sperm DNA damage risk who may benefit from antioxidant therapy or advanced sperm selection [12,13] et (iiii) reflect treatment response as a dynamic biomarker in personalized follow-up [9].

Given the growing emphasis on precision andrology, cfDNA may therefore play a pivotal role in stratifying male infertility severity.

### 4.4. Strengths and Limitations

The major strength of this study lies in its rigorous phenotypic classification using WHO 2021 standards [15], combined with standardized qPCR for RNase P gene quantification. Furthermore, this work provides data from a North African population, a region poorly represented in current cfDNA literature.

However, several methodological limitations warrant consideration. First, oxidative stress markers such as MDA or TAC were not evaluated, precluding a mechanistic link between cfDNA release and reactive oxygen species-induced damage. Second, genetic testing was not performed in azoospermic men, limiting the capacity to classify NOA subtypes with different pathophysiological substrates [1,16]. Third, ART outcomes were not included, which prevents assessing the prediction of fertilization or pregnancy success [13].

Future prospective multicenter studies should therefore incorporate oxidative stress profiling, genetic etiology, and live-birth-based outcomes to fully validate the clinical utility of cfDNA as a diagnostic and prognostic biomarker.

Altogether, this study confirms that seminal cfDNA is significantly elevated in the most severe forms of spermatogenic impairment, especially azoospermia and very severe teratozoospermia. By reflecting both testicular integrity and nuclear remodeling efficiency, cfDNA offers added value to conventional semen parameters and opens new perspectives in precision reproductive medicine, supporting optimized clinical management and personalized ART pathways.

## 5. Conclusions

This study demonstrates that seminal cfDNA levels are significantly elevated in men with severe sperm abnormalities, particularly azoospermia and severe teratozoospermia, supporting its potential value as a non-invasive molecular biomarker for the assessment of male infertility. By complementing conventional semen analysis, cfDNA quantification could improve diagnostic precision, guide therapeutic choices, and help identify patients who may require advanced ART strategies.

Future investigations should focus on standardizing analytical protocols and establishing clinically relevant cfDNA thresholds to enhance diagnostic and prognostic utility. Large, multicenter studies including diverse populations and longitudinal follow-up are also needed to validate the generalizability of these findings and determine the impact of cfDNA on ART outcomes, particularly live birth rates and offspring health. Finally, interventional studies examining whether targeted therapies such as antioxidant or anti-inflammatory treatments can reduce cfDNA levels would offer valuable insight into its role as a biomarker for monitoring treatment response.

Collectively, this work contributes to advancing cfDNA as a promising tool for precision reproductive medicine, supporting more personalized and effective clinical management of male infertility.

## Figures and Tables

**Table 1:** Characteristics of patients’ semen.

Setting	Normozoospermia (*n* = 25)	Azoospermia (*n* = 25)	Severe Asthenozoospermia (*n* = 25)	Severe Oligozoospermia (*n* = 25)	Severe Teratozoospermia (*n* = 25)
Age (years)	32.93 ± 1.55	35.15 ± 1.01 (ns)	33.83 ± 1.55 (ns)	31.61 ± 1.45 (ns)	37.14 ± 0.55 (ns)
Volume (mL)	3.41 ± 1.25	1.90 ± 0.54 *	2.84 ± 0.96 (ns)	1.54 ± 0.46 *	2.08 ± 0.79 *
Count (×10^6^/ejaculate)	48.56 ± 2.34	0.08 ± 0.19 *	32.32 ± 2.59 (ns)	2.26 ± 0.31 *	41.64 ± 1.80 (ns)
Total motility (%)	48.07 ± 1.02	0.00 *	14.43 ± 9.35 *	46.94 ± 1.26 (ns)	43.80 ± 1.03 (ns)
Vitality (%)	61.02 ± 2.70	0.00 *	57.07 ± 0.87 *	55.39 ± 0.03 *	55.85 ± 0.91 *
Normal morphology (%)	5.30 ± 1.30	0.00 *	5.30 ± 1.30 (ns)	5.07 ± 0.92 (ns)	2.07 ± 1.33 *

Notes: Data expressed as the mean ± SD. Statistical comparison only vs. control group (Mann–Whitney U); * *p* < 0.05; ns = not significant (*p* ≥ 0.05); Spermatic parameters were assessed according to the WHO 2021 criteria

**Table 2:** Seminal cfDNA concentrations across study groups and significance after multiple comparison correction.

Groups	cfDNA (µg/mL) Moy. ± SD	Comparison vs. Control	*p*-Value	Significant After Bonferroni (α = 0.0125)
Normozoospermia (*n* = 25)	1.96 ± 0.27	–	–	–
Azoospermia (*n* = 25)	5.47 ± 1.17	↑	<0.001	yes
Severe asthenozoospermia (*n* = 25)	2.04 ± 0.21	↑	0.339	No
Severe oligozoospermia(*n* = 25)	2.11 ± 0.21	↑	0.046	No
Severe teratozoospermia (*n* = 25)	3.25 ± 1.21	↑	<0.001	yes

Notes: Values are presented as the mean ± standard deviation (SD). ↑ vs. Control indicates higher cfDNA levels compared with the normozoospermic group. Statistical significance was set at *p* < 0.05. cfDNA: cell-free DNA; SD: standard deviation.

**Table 3:** Effect size estimates: mean cfDNA differences vs. controls with 95% CI and adjusted *p*-values.

Groups	Mean ± SD (µg/mL)	Difference vs. Control (IC95%)	p Raw	p Holm	Conclusion
Azoospermia	5.47 ± 1.17	+3.51 (2.90–4.12)	<0.001	<0.003	Significatif
Very severe teratozoospermia	3.25 ± 1.21	+1.29 (0.71–1.87)	<0.001	<0.003	Significatif
Very severe oligozoospermia	2.11 ± 0.21	+0.15 (–0.01–0.31)	0.046	0.092	NS
Very severe asthenozoospermia	2.04 ± 0.21	+0.08 (–0.08–0.24)	0.339	0.339	NS

Only azoospermia and severe teratospermia maintain significance after adjustment.

## Data Availability

The original contributions presented in this study are included in the article. Further inquiries can be directed to the corresponding author.

## Abbreviations

The following abbreviations are used in this manuscript:

ART	Assisted Reproductive Technology
CFDNA	Cell-free DNA
DNA	Deoxyribonucleic Acid
ICSI	Intracytoplasmic sperm micro-injection
PGD	Preimplantation genetic diagnosis
MAP	Medically assisted reproduction
qPCR	Quantitative Polymerase Chain Reaction
